# Exogenous Iron Induces Mitochondrial Lipid Peroxidation, Lipofuscin Accumulation, and Ferroptosis in H9c2 Cardiomyocytes

**DOI:** 10.3390/biom14060730

**Published:** 2024-06-19

**Authors:** Konstantin G. Lyamzaev, He Huan, Alisa A. Panteleeva, Ruben A. Simonyan, Armine V. Avetisyan, Boris V. Chernyak

**Affiliations:** 1 Belozersky Institute of Physico-Chemical Biology, Lomonosov Moscow State University, 119991 Moscow, Russia; hehuan163@gmail.com (H.H.); jalice@yandex.ru (A.A.P.); rubsim@mail.ru (R.A.S.); avetis@belozersky.msu.ru (A.V.A.); 2 The Russian Clinical Research Center for Gerontology, Ministry of Healthcare of the Russian Federation, Pirogov Russian National Research Medical University, 117997 Moscow, Russia

**Keywords:** ferroptosis, mitochondria, lipofuscin, lipid peroxidation, mitochondria-targeted antioxidants

## Abstract

Lipid peroxidation plays an important role in various pathologies and aging, at least partially mediated by ferroptosis. The role of mitochondrial lipid peroxidation during ferroptosis remains poorly understood. We show that supplementation of exogenous iron in the form of ferric ammonium citrate at submillimolar doses induces production of reactive oxygen species (ROS) and lipid peroxidation in mitochondria that precede ferroptosis in H9c2 cardiomyocytes. The mitochondria-targeted antioxidant SkQ1 and the redox mediator methylene blue, which inhibits the production of ROS in complex I of the mitochondrial electron transport chain, prevent both mitochondrial lipid peroxidation and ferroptosis. SkQ1 and methylene blue also prevented accumulation of lipofuscin observed after 24 h incubation of cardiomyocytes with ferric ammonium citrate. Using isolated cardiac mitochondria as an in vitro ferroptosis model, it was shown that rotenone (complex I inhibitor) in the presence of ferrous iron stimulates lipid peroxidation and lipofuscin accumulation. Our data indicate that ROS generated in complex I stimulate mitochondrial lipid peroxidation, lipofuscin accumulation, and ferroptosis induced by exogenous iron.

## 1. Introduction

Iron is an essential element crucial for the proper functioning of numerous life processes. It is an integral component of various proteins, forming complexes with the heme porphyrin ring or in iron–sulfur (Fe–S) clusters [1]. These proteins play important roles in oxygen transport, respiration coupled to ATP production, DNA synthesis and repair, and intracellular signaling. However, the presence of free reduced iron ions (Fe^2+^) poses a potential threat, as they catalyze the Fenton reaction, generating reactive oxygen radicals that can damage lipids, proteins, and DNA. Thus, maintaining precise control of redox-active iron in the labile iron pool (LIP) through regulation of iron transport, storage, and intracellular distribution is of paramount importance.

In plasma, iron in the oxidized form (Fe^3+^) binds tightly to transferrin, which facilitates its delivery into cells upon binding to the transferrin receptor (TRP1) and subsequent internalization through clathrin-mediated endocytosis [2]. Once inside endosomes, iron is released from transferrin due to acidic pH, reduced to Fe^2+^ by ferrireductases, and then transported into the cytosol through various divalent metal transporters [3]. Additionally, iron, in conjunction with other carrier proteins, undergoes receptor-mediated endocytosis.

Intracellular uptake of free iron involves ferrireductases catalyzing reduction to Fe^2+^. Within the cytosol, Fe^2+^ is predominantly retained in complexes with ferritin. Several chaperones play an important role in delivering iron to ferritin and other cytosolic iron-containing proteins, such as prolyl hydroxylases [4]. These chaperones also facilitate iron delivery to ferroportin (FPN) [5], the primary exporter of iron from cells, whose activity is regulated by the major iron-regulating hormone, hepcidin [6].

Mitochondria are major consumers of iron for synthesizing heme and Fe–S clusters, which are used to form hemoproteins and Fe–S proteins within mitochondria or exported to the cytosol. Fe–S clusters play critical roles in mitochondrial electron transport chain complexes I and II, as well as in heme synthesis as components of δ-aminolevulinate dehydratase (ALAD) [7] and ferrochelatase (FECH) [8]. Hemes, essential components of cytochromes in mitochondrial complexes III and IV, also contribute to intracellular signal transduction through proteins like nitric oxide synthase (NOS) [9]. The transport of iron into mitochondria involves specific transporters such as divalent metal transporter DMT1 [10] in the outer mitochondrial membrane and mitoferrins (MFRN1 and MFRN2) in the inner membrane [11,12]. Mitochondria house a distinct isoform of ferritin [13], and the regulation of free iron in the mitochondrial matrix parallels that in the cytosol. Mitochondrial iron export primarily depends on ATP-binding cassette (ABC) transporters [14]. Interestingly, it was reported recently that oxidative stress induced by organic hydroperoxides promotes expression of heme oxygenase 1 (HO-1) and its translocation to mitochondria, leading to mitochondrial iron overload [15]. The mechanisms of mitochondrial, cellular, and systemic iron homeostasis have recently been comprehensively reviewed [16].

Dysregulation of iron homeostasis contributes to various disorders, including metabolic disorders, inflammatory diseases, heart failure, chronic liver diseases, neurodegeneration, and more. Systemic iron overload can result from mutations in genes regulating hepcidin or cellular iron uptake during intestinal absorption (primary hemochromatosis) [17], due to impaired erythropesis (β-thalassemia, sickle cell disease), rhabdomyolysis (crush syndrome) or multiple blood transfusions, for example, in hematopoietic stem cell transplant recipients [18]. Ischemia/reperfusion, oxidative stress, and inflammation disrupt cellular iron homeostasis, leading to iron overload and damage to the liver [19], heart [20], and kidneys [21], as well as neurodegenerative diseases [22]. Ferroptosis, an iron-dependent form of regulated cell death, appears to be implicated in these disorders [23]. Nonenzymatic lipid peroxidation catalyzed by Fe^2+^ is a critical event in ferroptosis [24]. Ferroptosis can be caused by cystine starvation, the cystine uptake inhibitor erastin, inhibition of glutathione biosynthesis, inhibitors of glutathione peroxidase 4 (GPx4), which detoxifies lipid peroxides, or by exogenous iron [23].

Recent research emphasizes the crucial role of mitochondrial iron in conditions associated with iron overload. Overexpression of mitochondrial ferritin has demonstrated protective effects against hypoxia-related cardiac [25] and brain pathologies [26]. The involvement of mitochondria in ferroptosis has been a subject of debate, initially stemming from Stockwell and colleagues’ pioneering work [24], which showed that mitochondrial DNA depletion had no significant impact on ferroptosis sensitivity. However, subsequent studies utilizing more suitable models failed to confirm these results [27,28]. Evidence supporting the role of mitochondrial reactive oxygen species (ROS) production in ferroptosis comes from studies using mitochondria-targeted antioxidants such as XJB-5-131 [29] and MitoQ [28]. Additionally, the mitochondria-targeted antioxidant MitoTEMPO has been shown to prevent doxorubicin-induced cardiac ferroptosis in mice [30]. In our recent investigation employing a novel lipid peroxidation-sensitive fluorescent probe MitoCLox, we established that mitochondrial lipid peroxidation (LPO) precedes cell death induced by erastin or glutathione depletion [31]. The critical role of mitochondrial LPO in these models of ferroptosis is supported by data showing that the mitochondria-targeted antioxidants SkQ1 (10-(6′-plastoquinonyl) decyltriphenylphosphonium bromide) and MitoTEMPO inhibit both LPO and ferroptosis.

Beyond ferroptosis, recent discoveries indicate that iron overload can induce cellular senescence and the senescence-associated secretory phenotype (SASP) [32]. While ROS are recognized as major contributors to cellular senescence [33], the specific role of mitochondrial ROS (mtROS) production remains unclear. It has been suggested that mtROS may cause DNA breaks in telomeric regions and activate signaling pathways associated with cell cycle arrest [34]. Surprisingly, the potential involvement of iron-dependent mitochondrial lipid peroxidation in cellular senescence has not been thoroughly explored. Meanwhile, lipid peroxidation products are known to contribute to the formation of lipofuscin, a recognized marker of cellular senescence [35].

The accumulation of lipofuscin, a heterogeneous complex mixture primarily composed of highly oxidized lipids and cross-linked proteins can interfere with both the proteasomal and lysosomal degradation systems, potentially accelerating senescence. Notably, lipofuscin has been reported to catalyze ROS formation in senescent cells by binding redox-active iron to its surface [36]. Early investigations have identified mitochondria and lysosomes as the main contributors to lipofuscin [37], with mitochondrial lipofuscin formation implicated in the “mitochondrial-lysosomal axis theory of aging” [38]. However, the mechanisms underlying lipofuscin formation in mitochondria remain unclear. It is important to note that lipofuscin accumulation, possibly associated with iron overload, can occur independently of cell proliferation, especially in postmitotic cells such as neurons and cardiomyocytes [38].

In our current study, we show that exogenous iron supplemented in the form of ferric ammonium citrate at submillimolar doses induces production of ROS and lipid peroxidation in mitochondria that precede ferroptosis in H9c2 cardiomyocytes. The mitochondria-targeted antioxidant SkQ1 and the redox mediator methylene blue exhibit inhibitory effects on both lipid peroxidation and ferroptosis, along with a reduction in lipofuscin accumulation. Using isolated cardiac mitochondria as an in vitro ferroptosis model, it was demonstrated that ROS generated by complex I of the mitochondrial electron transport chain stimulate iron-dependent lipid peroxidation and lipofuscin formation.

## 2. Materials and Methods

### 2.1. Chemicals

SkQ1 and dodecyltriphenylphosphonium bromide (C_12_TPP) were kindly provided by the Institute of Mitoengineering, Lomonosov Moscow State University. C11-BODIPY581/591 was from Lumiprobe (Moscow, Russia). CM-H2DCFDA and MitoSOX were from Invitrogen Life Technologies (Waltham, MA, USA). MitoCLox was synthesized from succinimidyl ester of C11-BODIPY581/591 and (5-[(4-aminobutyl)amino]-5-oxopentyl) triphenylphosphonium bromide as described in [39]. MitoCLox, a ratiometric fluorescent dye that specifically reacts with lipid peroxy radicals, is addressed to mitochondria by conjugation of the fluorophore C11-BODIPY581/591 with the penetrating triphenylphosphonium cation. MitoCLox was shown to selectively accumulate in the mitochondria of various living cells and registers mitochondrial lipid peroxidation [40,41]. Other reagents, except for those indicated, were from Sigma-Aldrich (Saint Louis, MO, USA).

### 2.2. Cell Cultures

Rat cardiomyocytes H9c2, a spontaneously immortalized cell line (EcACC Cat. No. 88,092,904), and primary skin fibroblasts from Common Use Center “Biobank” (Research Centre for Medical Genetics, Moscow, Russia) were cultured in Dulbecco’s modified Eagle’s medium (DMEM) (Gibco; Thermo Fisher Scientific, Inc., Waltham, MA, USA) supplemented with 2 mM glutamine and 10% fetal bovine serum (FBS) (HyClone, Logan, UT 84321 USA) and 100 U/mL streptomycin and 100 U/mL penicillin (all from Gibco, USA). Cell viability was measured using the CellTiterBlue^®^ reagent (Promega, Madison, WI, USA) according to the manufacturer’s protocol with Fluoroskan Ascent FL Microplate Reader (Thermo Labsystems, Waltham, MA, USA).

### 2.3. Microscopy

H9c2 cells were plated in 35 mm glass-bottom (SPL) dishes for confocal microscopy at 150,000 cells. After incubation with ferric ammonium citrate (FAC) for 24 h, cells were stained with 50 μg/mL propidium iodide and 8 μM Hoechst 33,258 for 30 min. Image acquisition was performed using a fluorescence microscope (Olympus IX 83, Tokyo, Japan).

### 2.4. Flow Cytometry

H9c2 cells or primary human fibroblasts were stained with 100 nM MitoCLox (1 h) or 2 μM C11-BODIPY581/591 (30 min), 1.8 µM CM-H2DCFDA (30 min), and 1 µM MitoSOX (30 min). Cells were stripped with trypsin/versene, centrifuged in 1.5 mL tubes (900 g, 5 min) at 4 °C, and redispersed in 50 μL PBS. Flow cytometry analyses were performed using an Amnis FlowSight Imaging Flow Cytometer (Luminex Corporation, Seattle, WA, USA) with excitation at 488 nm and the detection channels 480–560 nm (Ch2) and 595–642 nm (Ch4). Channel 2 (Ch2) was used to detect autofluorescence, which indicates lipofuscin content [42]. Each sample was measured until 4000 events were collected. For ratiometric analysis, the Amnis IDEAS^®^ 6.2 (Luminex, Seattle, WA, USA) image analysis software was used. In cases where the signal distribution was far from Gaussian, a gating procedure was used to evaluate the effects.

### 2.5. Isolated Mitochondria

Mitochondria were isolated from rat hearts in the medium containing 250 mM sucrose, 5 mM Mops KOH, pH 7.4, 1 mM EGTA, and bovine serum albumin (0.5 mg/mL). The heart was minced with scissors in a cooled isolation medium in a ratio of 10 mL/g of cardiac tissue and homogenized in a glass Potter homogenizer for 1–2 min. The homogenate was diluted with an isolation medium to the ratio of 20 mL/g of original tissue and centrifuged for 10 min at 600× *g*. The supernatant was collected and centrifuged for 10 min at 12,000× *g*. The precipitate was resuspended in a minimum volume, homogenized, diluted in 20 mL of isolation medium, and centrifuged for 10 min at 12,000× *g*. The pellet was resuspended and stored on ice. The lipid peroxidation was measured in the medium containing 250 mM sucrose, 5 mM Mops KOH, pH 7.4, and 50 nM MitoCLox at 25 °C. For analysis of lipofuscin accumulation, mitochondria in the same medium (except MitoCLox) were incubated for 24 h at 37 °C and 0.5% SDS was added before measurements. Additions: 10–100 µM FeSO_4_, 5 mM glutamate/5 mM malate (G/M), 10 µM rotenone, 5 mM succinate, and 2 µM antimycin A. The concentration of mitochondrial protein was 0.4 mg/mL, as determined by Bradford method. Fluorescence was measured using FluoroMax-3 spectrofluorometer (Horiba Sci., Kyoto, Japan).

### 2.6. Statistics

At least three repeats for each measurement were performed. Results are presented as the mean of a minimum of 3 independent replicates with standard deviation (SD). Comparisons were analyzed by one-way ANOVA. The significance was analyzed with Prism 10.0 software (GraphPad Software, LLC, Solana Beach, CA, USA); a value of *p* < 0.05(^#^,*) was considered to be statistically significant.

## 3. Results

### 3.1. Exogenous Iron Added as Ferric Ammonium Citrate Induces Ferroptosis in H9c2 Cardiomyocytes

**Figure 1:**
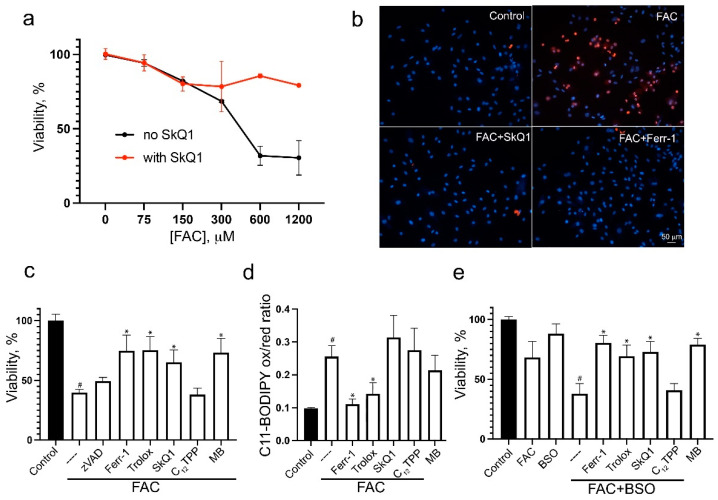
Ferric ammonium citrate induces ferroptosis in H9c2 cardiomyocytes. (**a**) Cells were incubated with FAC for 48 h. Cell viability was measured using the CellTiterBlue reagent. A total of 50 nM SkQ1 (red points) was added simultaneously with FAC. (**b**) Cells were incubated with 0.6 mM FAC alone and in combination with 50 nM SkQ1 or 0.1 mM Ferr-1 for 48 h, stained with 50 μg/mL propidium iodide (red) and 8 μM Hoechst 33,258 (blue) for 30 min, and analyzed using Olympus IX83. Bar 50 μm. (**c**) Cells were incubated with 0.6 mM FAC alone and in combination with 10 μM zVADfmk (zVAD), 0.1 mM Ferr-1, 50 nM SkQ1, 50 nM C_12_TPP, and 250 nM MB for 48 h. (**d**) Cells were incubated with 0.6 mM FAC alone and in combination with 50 nM SkQ1, 50 nM C_12_TPP, 0.1 mM Ferr-1, 0.2 mM Trolox, and 250 nM MB for 24 h. Then, cells were stained with 2 μM C11-BODIPY581/591 for 30 min and analyzed using flow cytometry. Ratio of green/red fluorescence was measured. Mean values are presented. (**e**) Cells were incubated with 0.6 mM FAC alone and in combination with 1 mM BSO for 48 h. A total of 50 nM SkQ1, 50 nM C_12_TPP, 0.1 mM Ferr-1, 0.2 mM Trolox, and 250 nM MB were added simultaneously with FAC and BSO. *p* < 0.05 (#)—the significance of the difference between control and FAC or FAC+BSO sample. *p* < 0.05 (*)—the significance of the difference between samples treated with FAC or FAC+BSO and other samples.

To model iron overload in vitro, we used ferric ammonium citrate (FAC), which is a physiological form of nontransferrin-bound iron widely used as a dietary supplement. In the pioneering work of Dixon et al. [24], FAC was used as a sensitizer in erastin-induced ferroptosis of HT-1080 fibrosarcoma cells. These cells are highly sensitive to the induction of ferroptosis and are often used as a model to investigate the mechanisms of ferroptosis; however, FAC has been shown to kill these cells only at extremely high concentrations (5 mM and more) [43]. Quite unexpectedly, we observed that FAC reduced the viability of H9c2 cardiomyocytes at submillimolar concentrations (Figure 1a). FAC-induced cell death was necrotic, as revealed by propidium iodide staining of nuclei (Figure 1b), and was not prevented by the pan-caspase inhibitor zVADfmk (Figure 1c), so secondary caspase-dependent necrosis was excluded. The ferroptosis inhibitor ferrostatin-1 (fer-1) and another antioxidant Trolox (a water-soluble vitamin E analogue) completely prevented the FAC-dependent decrease in viability (Figure 1c). Finally, measurements of lipid peroxidation using the BODIPY-C11581/591 dye show that LPO precedes FAC-dependent cell death and is blocked by fer-1 and Trolox (Figure 1d). Thus, all these data indicate that FAC induces ferroptosis in H9c2 cells.

The FAC-induced decrease in H9c2 cell viability was strongly promoted by the gamma-glutamyl cysteine synthetase inhibitor buthionine sulfoximine (BSO), which caused glutathione depletion but was not toxic to H9c2 cells down to millimolar concentrations (Figure 1e). Necrotic cell death in this case was prevented by Fer-1 and Trolox, indicating ferroptosis. These data suggest that cell death caused by iron overload is counterbalanced by glutathione-dependent protective mechanisms.

Ferroptosis induced either by FAC alone or in combination with BSO was prevented by the mitochondria-targeted antioxidant SkQ1, whereas the SkQ1 analog lacking the antioxidant moiety C_12_TPP was ineffective (Figure 1a,c,e). Ferroptosis was also inhibited by the redox cycling agent methylene blue (MB), which targets mitochondria due to its positive charge and bypasses electron flow past complex I of electron transport chain (ETC) [44,45]. Thus, FAC-induced ferroptosis depends on the production of mitochondrial ROS (mtROS), which is originated, at least in part, from complex I.

### 3.2. FAC-Induced Ferroptosis Depends on Mitochondrial ROS Production and Lipid Peroxidation

**Figure 2:**
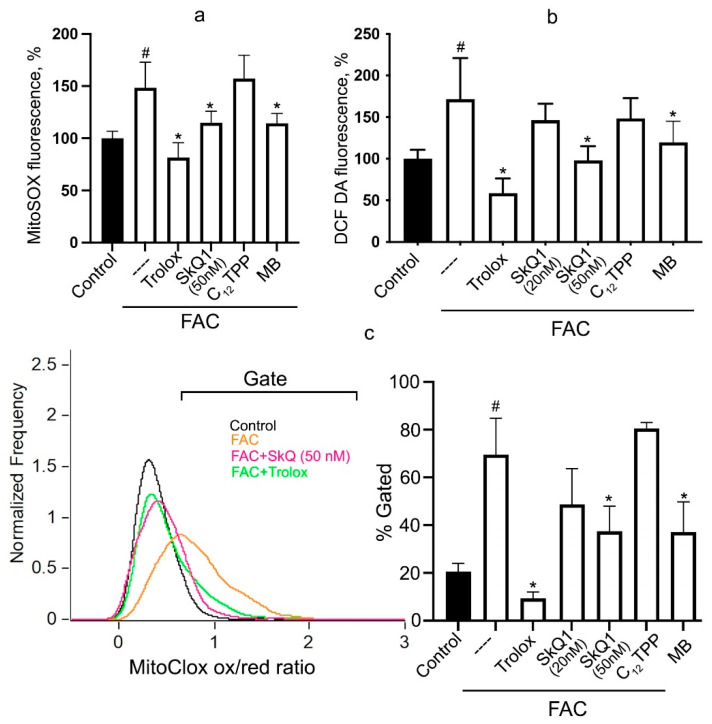
Ferrous ammonium citrate induces oxidative stress, ROS production, and lipid peroxidation in mitochondria. Cells were incubated with 0.6 mM FAC alone and in combination with 20 nM or 50 nM SkQ1, 50 nM C_12_TPP, 250 nM MB, or 0.2 mM Trolox for 24 h and analyzed using flow cytometry. (**a**) Cells were stained with 1 µM MitoSOX for 30 min. Mean values of fluorescence are presented. (**b**) Cells were stained with 1.8 µM CM-H2DCFDA for 30 min. Mean values of fluorescence are presented. (**c**) Cells were stained with 100 nM MitoCLox for 1 h. Ratio of green/red fluorescence was measured and analyzed using gating procedure. *p* < 0.05 (#)—the significance of the difference between control and FAC sample. *p* < 0.05 (*)—the significance of the difference between samples treated with FAC and other samples.

FAC induced a significant increase in mitochondrial ROS (mtROS) production, as revealed by mitochondria-targeted dye MitoSOX (Figure 2a). This effect was inhibited by either Trolox or by SkQ1 at much lower concentrations. Importantly, mtROS production was also inhibited by MB in full agreement with the data on cell viability (Figure 1).

The FAC-induced lipid peroxidation in mitochondria was analyzed using a new mitochondria-targeted fluorescent ratiometric probe MitoCLox [39]. The specific oxidation of MitoCLox by lipid radicals, its selective accumulation in mitochondria of various cells, and the response to mitochondrial LPO was shown earlier [40,41]. This probe was used in our previous study to demonstrate mitochondrial LPO during erastin-induced ferroptosis and ferroptosis induced by butionine sulfoximine in fibroblasts from patient with Leber’s hereditary optic neuropathy (LHON) [31]. Incubation with FAC for 24 h induced significant oxidation of MitoCLox in a fraction of the cells and the level of mitochondrial LPO was highly heterogenous in this cell fraction (Figure 2c). The similar heterogeneity of mitochondrial LPO was also observed in two models of ferroptosis described earlier [31]. SkQ1, but not C_12_TPP, as well as methylene blue and Trolox prevent FAC-induced mitochondrial LPO (Figure 2c). SkQ1 and methylene blue strongly suppressed FAC-induced cell death (Figure 1), indicating that mtROS production and mitochondrial LPO are critical for ferroptosis.

Cytosolic hydrogen peroxide accumulation, measured by CM-H2DCFDA, was significantly increased after 24 h incubation with FAC (Figure 2b). SkQ1 and MB prevent FAC-induced H_2_O_2_ accumulation, indicating that mtROS contribute significantly to the induction of general oxidative stress in this model. Interestingly, the same analysis did not reveal the effect of SkQ1 on erastin-induced H_2_O_2_ accumulation in fibroblasts [31], indicating that the contribution of mtROS to overall oxidative stress differs in different models of ferroptosis, while mitochondrial lipid peroxidation remains critical in all studied models.

FAC-induced total lipid peroxidation measured by C11-BODIPY581/591 at 24 h was not affected by SkQ1 or MB, whereas it was prevented by the nontargeted antioxidants ferrostatin-1 and Trolox (Figure 1d). These data indicate that LPO, which develops in all cell membranes, cannot always be considered as a marker of ferroptosis. In our model, the FAC-induced LPO observed in the presence of SkQ1 or MB was not accompanied by cell death. The contribution of mtROS and mitochondrial lipid peroxidation to total lipid peroxidation induced by FAC turned out to be insignificant.

In addition, we examined the effects of the first-in-line inducer of ferroptosis erastin in H9c2 cells (Supplementary Figure S1). It has been shown that erastin dose-dependently induces necrotic cell death, which is preceded by mitochondrial lipid peroxidation and accumulation of hydrogen peroxide in the cytosol. SkQ1 and MB inhibit both mitochondrial and cytosolic events and protect against ferroptosis, indicating that mitochondria contribute significantly to oxidative stress and the induction of ferroptosis caused either by inhibition of glutathione production (erastin) or by iron overload (FAC) in cardiomyocytes.

### 3.3. FAC Induces Rapid Accumulation of Lipofuscin-Like Material in H9c2 Cells

Incubation of H9c2 with FAC for 24 h leads to a significant accumulation of lipofuscin-like material, as evidenced by an increase in autofluorescence in a wide spectral range of 480–560 nm. Lipofuscin is considered a marker of organismal aging and cellular senescence. In vivo, lipofuscin accumulates in cardiomyocytes very slowly. In the rat heart, its content increases linearly during development and aging (from 5 to 14 and from 14 to 24 months) [46]. In the heart of long-lived large animals, lipofuscin accumulates much more slowly than in small short-lived animals [47]. The very rapid accumulation of lipofuscin-like material induced by FAC presumably reflects active iron-dependent lipid peroxidation in H9c2 cells.

**Figure 3:**
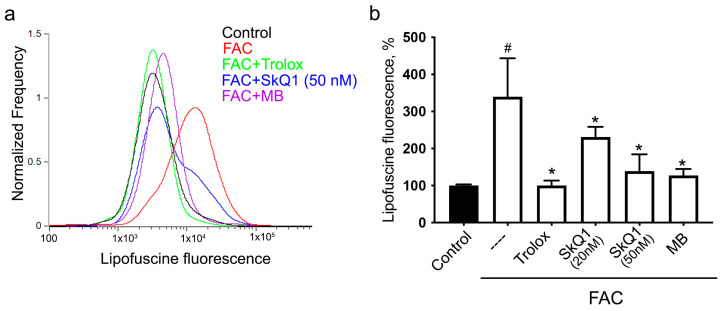
Ferrous ammonium citrate induces accumulation of lipofuscin-like material in H9c2 cells. Cells were incubated with 0.6 mM FAC alone and in combination with 20 nM or 50 nM SkQ1, 50 nM C_12_TPP, 250 nM MB, or 0.2 mM Trolox for 24 h and analyzed using flow cytometry without staining. Representative histograms (**a**) and mean values of fluorescence (**b**) are presented. *p* < 0.05 (#)—the significance of the difference between control and FAC sample. *p* < 0.05 (*)—the significance of the difference between samples treated with FAC and other samples.

FAC-induced accumulation of lipofuscin-like material was prevented by SkQ1 and MB as well as by Trolox (Figure 3), indicating that mtROS production and mitochondrial LPO are critical. Since oxidized lipids are an important component of lipofuscin, it can be assumed that mitochondrial lipids are directly involved in the formation of lipofuscin.

### 3.4. Iron-Dependent Lipid Peroxidation and Accumulation of Lipofuscin-Like Material in Isolated Mitochondria

**Figure 4:**
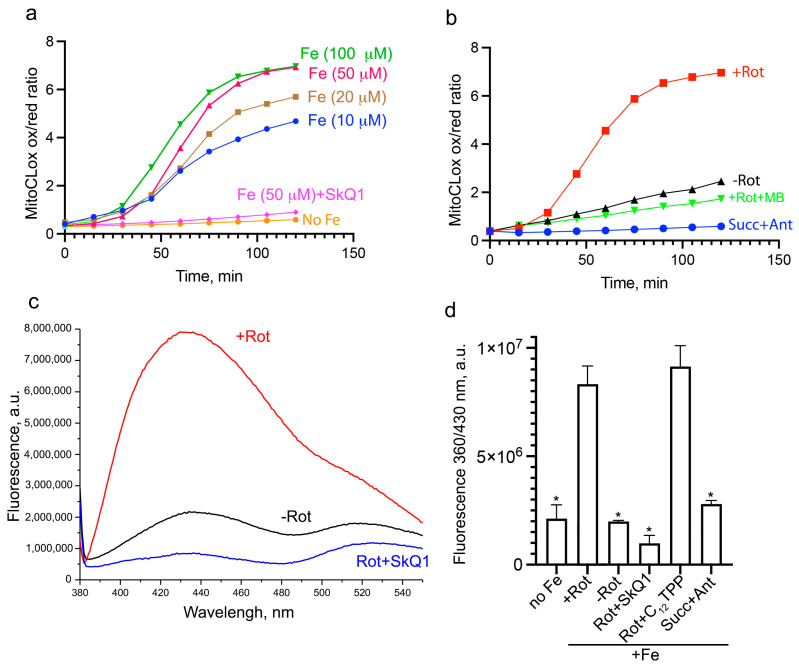
Mitochondrial lipid peroxidation (**a**,**b**) and accumulation of lipofuscin-like material (**c**,**d**) induced by Fe^2+^ in isolated rat heart mitochondria. (**a**,**b**) Mitochondria were incubated with 50 nM MitoCLox and various concentrations of FeSO_4_ in the presence of glutamate (5 mM), malate (5 mM), rotenone (rot, 10 μM), or in the presence of succinate (succ, 5 mM) and antimycine A (Ant, 1 μM). A total of 300 nM SkQ1, 300 nM C_12_TPP, or 1 μM MB were added simultaneously with 50 μM FeSO_4_. Where indicated, rotenone was omitted or succinate and antimycine A were added. In the probe “no Fe”, 0.1 mM EGTA was added. Fluorescent spectra were analyzed after subtraction of light scattering. Kinetics of MitoCLox oxidation calculated as a ratio of fluorescence at 521 nm and 596 nm are shown. (**c**,**d**) Mitochondria were incubated with 50 μM FeSO_4_ in the presence of glutamate (5 mM), malate (5 mM), rotenone (10 μM), or in the presence of succinate (succ, 5 mM) and antimycine A (1 μM, Ant) for 40 min or 24 h at 37 °C and destructed with 0.5% SDS. A total of 300 nM SkQ1 or 300 nM C_12_TPP were added simultaneously with FeSO_4_. In the probe “no Fe”, 0.1 mM EGTA was added in the presence of glutamate (5 mM), malate (5 mM), and rotenone (10 μM). Fluorescent spectra at 360 nm excitation were analyzed. The representative spectra obtained by subtracting the 40 min spectra from the corresponding 24 h spectra (**c**) and fluorescence at 430 nM from these spectra (**d**) are shown. *p* < 0.05 (*)—the significance of the difference between samples treated with Rot+Fe and other samples.

In our previous study [31], we analyzed lipid peroxidation in isolated rat heart mitochondria using MitoCLox. It has been shown that LPO is observed only in the presence of ferrous ion (Fe^2+^), and the addition of the complex I inhibitor rotenone in the presence of NAD-dependent substrates (glutamate and malate) strongly stimulates oxidation. Using the same assay, we studied the dependence of mitochondrial LPO on FeSO_4_ concentration (Figure 4a). It was shown that the rate of oxidation increases with increasing FeSO_4_ concentration to 50 µM, while further acceleration with increasing concentration to 200 µM is less pronounced. Presumably, at FeSO_4_ concentrations above 50 µM, the rate of lipid peroxidation is limited by other factors. It is important to note that, even at 50 μM FeSO_4_, LPO was strongly stimulated by rotenone and prevented by SkQ1 (Figure 4).

We have previously shown [31] that the complex III inhibitor antimycin A does not induce lipid peroxidation in the presence of succinate, although the rate of hydrogen peroxide production in this case was even higher than in the presence of glutamate, malate, and rotenone. The same picture was observed at 50 μM FeSO_4_ (Figure 4b). It can be assumed that the production of ROS on the flavin mononucleotide (FMN) of complex I (excessively reduced in the presence of rotenone), and not the ROS produced by complex III, stimulates lipid peroxidation at high Fe^2+^ concentrations. This conclusion is fully consistent with the inhibition of lipid peroxidation by MB that bypass electron flow past complex I (Figure 4b).

We used isolated mitochondria to study iron-induced accumulation of lipofuscin-like material. Lipofuscin accumulates in isolated mitochondria incubated in the presence of 50 μM FeSO_4_, glutamate, malate, and rotenone for 24 h at 37 °C (Figure 4c). Much less accumulation was observed in the absence of rotenone. Very low accumulation of lipofuscin was caused by antimycin A and succinate. SkQ1 inhibits the accumulation of lipofuscin in those doses that are necessary for the prevention of lipid peroxidation (Figure 4c,d). It was not possible to use MB in these long-term experiments due to its low stability during continuous redox cycling. These data confirm our assumption about the direct participation of oxidized mitochondrial lipids in the formation of lipofuscin.

## 4. Discussion

The role of iron overload and ferroptosis in various pathologies is well established [15,22], which makes it surprising that there is a large gap in understanding the mechanisms of ferroptosis caused by exogenous iron. This reflects the very low toxicity of iron added in the physiological form of ferric ammonium citrate to cells, widely used in ferroptosis research. Thus, it was shown that, in HT-1080 fibrosarcoma cells, which are highly sensitive to the induction of ferroptosis by erastin or Gpx4 inhibitors, FAC induces ferroptosis only at concentrations of 5 mM and higher [43]. Even with 8 mM FAC, only a 40% decrease in cell viability was observed after 24 h incubation [48]. Similar doses of FAC have been shown to induce ferroptosis in oligodendrocytes [49] and non-small-cell lung carcinoma cell lines [50]. At the same time, FAC has been shown to stimulate ferroptosis induced by erastin [24] and some other inducers [51].

As shown in Figure 1, FAC induces ferroptosis in H9c2 cardiomyocytes at submillimolar doses. Importantly, FAC toxicity was strongly exacerbated by inhibition of glutathione biosynthesis by BSO (Figure 1e), indicating that glutathione-dependent protective (presumably antioxidant) mechanisms significantly limit ferroptosis induced by exogenous iron. Induction of ferroptosis by FAC in the same concentration range has been described in the mouse islet β-cell line MIN6 [52]. These observations are consistent with in vivo studies that have shown that iron overload can impair insulin secretion and is an important risk factor for the development of type 2 diabetes [53]. Another example of cells that are sensitive to exogenous iron is osteoblasts. FAC has been shown to induce osteoblast ferroptosis in vitro and induce osteoporosis in vivo [54].

FAC-induced ferroptosis is preceded by increased mitochondrial ROS production (Figure 2a) and mitochondrial lipid peroxidation (Figure 2c). Both oxidative events were prevented by the mitochondria-targeted antioxidants SkQ1 and the redox cycling agent methylene blue, which inhibits ROS formation in complex I of the mitochondrial ETC. Both SkQ1 and MB prevent cell death induced by FAC (Figure 1) as well as by erastin (Supplementary Materials Figure S1), suggesting that mtROS production and mitochondrial lipid peroxidation contribute significantly to the mechanism of ferroptosis induced by either exogenous iron or glutathione depletion.

In our previous study [31], using the same approaches, we demonstrated that mitochondrial lipid peroxidation is critical for ferroptosis induced by erastin in normal fibroblasts or BSO in fibroblasts from patients with Leber hereditary optic neuropathy. In contrast to these fibroblast-based models, overall oxidative stress induced by FAC in cardiomyocytes was suppressed by SkQ1 and MB, indicating a significant role for mitochondria. Probably, the high sensitivity of cardiomyocytes to exogenous iron is associated with the high content and metabolic activity of mitochondria in these cells.

In support of our conclusion about the critical role of mitochondrial oxidative events in ferroptosis, several mitochondria-targeted antioxidants such as MitoQ (ubiquinol-based SkQ1 analogue), MitoTEMPO (piperidine nitroxide TEMPO conjugated with triphenylphosphonium cation), or XJB-5-131 (TEMPO conjugated with gemigramicidin S) inhibit mitochondrial lipid peroxidation and ferroptosis induced by erastin or the Gpx4 inhibitor RSL3 [27,28,29], whereas their nontargeted analogues were much less effective. Additional support has come from studies of overexpression of the mitochondrial ferritin isoform (MtFt), which binds labile iron in the mitochondrial matrix, inhibiting the formation of hydroxyl radicals and lipid peroxidation. Overexpression of MtFt has been shown to significantly inhibit erastin-induced ferroptosis in SH-SY5Y neuroblastoma cells [55], as well as ferroptosis induced by doxorubicin or simulated ischemia/reperfusion in neonatal rat cardiomyocytes [15].

The products of lipid peroxidation can form a heterogeneous complex with oxidized cross-linked proteins, forming lipofuscin, which is a good marker of cellular senescence as well as organismal aging [35]. Autofluorescence measurements over a wide range of wavelengths revealed the accumulation of lipofuscin-like material during 24 h of incubation with FAC (Figure 3). It is known that this fluorescence mainly belongs to Schiff bases formed by aldehydes resulting from lipid peroxidation and amino groups of proteins [56]. Typical lipofuscin granules are formed in lysosomes as a result of the accumulation and incomplete degradation of cross-linked protein–lipid aggregates. At the same time, in a model of oxidative-stress-induced accelerated aging caused by paraquat, it was shown that lipofuscin-like material accumulates in the cytoplasm regardless of lysosomal activity [57]. Moreover, inhibition of macroautophagy and lysosomal proteolysis can accelerate lipofuscin accumulation both in vitro [57] and in vivo [58]. It can be assumed that the rapid formation of lipofuscin-like material during iron overload occurs due to strong lipid peroxidation, as well as impaired autophagy/lysosome functions.

FAC-induced accumulation of lipofuscin-like material was prevented by SkQ1 and MB (Figure 3). These data indicate that mtROS production and mitochondrial LPO are important for lipofuscin formation. In full agreement with our data, in a model of paraquat-induced senescence, the mitochondria-targeted antioxidant mitoTEMPO prevented the accumulation of lipofuscin [59]. Moreover, in this model, the mitochondrial fission and mitophagy inhibitor Mdivi-1 inhibited the formation of lipofuscin. Another approach to inhibiting mitophagy has been reported to suppress lipofuscin accumulation in hippocampal neurons during hypoxia [60].

To elucidate the potential role of mitochondria in the iron-dependent formation of lipofuscin, we studied this process in isolated mitochondria (Figure 4). Consistent with our previous studies [31], lipid peroxidation in isolated rat heart mitochondria is strongly dependent on Fe^2+^ concentration and on rotenone-stimulated production of ROS in complex I. Lipofuscin-like material accumulates under the same conditions during 24 h at 37 °C (Figure 4). SkQ1 inhibits lipofuscin accumulation in parallel with lipid peroxidation. The formation of lipofuscin in isolated mitochondria was observed as early as 1969 [37]. Later, the induction of lipofuscin formation by heat and UV light [61], as well as tert-butyl hydroperoxide and peroxynitrite [62], was described. Our data show for the first time that the formation of lipofuscin-like material can be catalyzed by mitochondrial lipid peroxidation caused by the production of ROS in mitochondria. These results suggest that lipid peroxidation in mitochondria can directly supply material for lipofuscin formation during iron overload.

## 5. Conclusions

In conclusion, we show that complex-I-dependent mitochondrial ROS production and lipid peroxidation are critical for ferroptosis induced by exogenous iron in cardiomyocytes. The rapid accumulation of lipofuscin-like material, indicative of accelerated aging, is also dependent on mitochondrial lipid peroxidation. Ferroptosis of cardiomyocytes [20] as well as premature aging are considered important factors mediating iron-overload-related cardiomyopathies. The mitochondria-targeted antioxidant SkQ1 effectively inhibits ferroptosis and lipofuscin formation caused by iron overload. Thus, mitochondria-targeted agents appear to be promising candidates for the treatment of various pathologies associated with iron overload.

## Data Availability

The datasets and raw data used and/or analyzed during the current study are available from the corresponding author on reasonable request.

## Supplementary Materials

The following supporting information can be downloaded at: https://www.mdpi.com/article/10.3390/biom14060730/s1. Figure S1: Erastin-induced ferroptosis in H9C2.

## References

[1] Andreini C., Putignano V., Rosato A., Banci L. (2018). The human iron-proteome. Metallomics.

[2] Cheng Y., Zak O., Aisen P., Harrison S.C., Walz T. (2004). Structure of the human transferrin receptor-transferrin complex. Cell.

[3] Luck A.N., Mason A.B. (2012). Transferrin-mediated cellular iron delivery. Curr. Top. Membr..

[4] Philpott C.C., Ryu M.S., Frey A., Patel S. (2017). Cytosolic iron chaperones: Proteins delivering iron cofactors in the cytosol of mammalian cells. J. Biol. Chem..

[5] Rishi G., Subramaniam V.N. (2021). Biology of the iron efflux transporter, ferroportin. Adv. Protein Chem. Struct. Biol..

[6] Nemeth E., Ganz T. (2023). Hepcidin and Iron in Health and Disease. Annu. Rev. Med..

[7] Liu G., Sil D., Maio N., Tong W.H., Bollinger J.M., Krebs C., Rouault T.A. (2020). Heme biosynthesis depends on previously unrecognized acquisition of iron-sulfur cofactors in human amino-levulinic acid dehydratase. Nat. Commun..

[8] Wu C.K., Dailey H.A., Rose J.P., Burden A., Sellers V.M., Wang B.C. (2001). The 2.0 A structure of human ferrochelatase, the terminal enzyme of heme biosynthesis. Nat. Struct. Biol..

[9] Zaobornyj T., Ghafourifar P. (2012). Strategic localization of heart mitochondrial NOS: A review of the evidence. Am. J. Physiol. Heart Circ. Physiol..

[10] Wolff N.A., Garrick M.D., Zhao L., Garrick L.M., Ghio A.J., Thevenod F. (2018). A role for divalent metal transporter (DMT1) in mitochondrial uptake of iron and manganese. Sci. Rep..

[11] Shaw G.C., Cope J.J., Li L., Corson K., Hersey C., Ackermann G.E., Gwynn B., Lambert A.J., Wingert R.A., Traver D. (2006). Mitoferrin is essential for erythroid iron assimilation. Nature.

[12] Paradkar P.N., Zumbrennen K.B., Paw B.H., Ward D.M., Kaplan J. (2009). Regulation of mitochondrial iron import through differential turnover of mitoferrin 1 and mitoferrin 2. Mol. Cell. Biol..

[13] Arosio P., Levi S. (2010). Cytosolic and mitochondrial ferritins in the regulation of cellular iron homeostasis and oxidative damage. Biochim. Biophys. Acta.

[14] Ichikawa Y., Bayeva M., Ghanefar M., Potini V., Sun L., Mutharasan R.K., Wu R., Khechaduri A., Jairaj Naik T., Ardehali H. (2012). Disruption of ATP-binding cassette B8 in mice leads to cardiomyopathy through a decrease in mitochondrial iron export. Proc. Natl. Acad. Sci. USA.

[15] Chen Y., Guo X., Zeng Y., Mo X., Hong S., He H., Li J., Fatima S., Liu Q. (2023). Oxidative stress induces mitochondrial iron overload and ferroptotic cell death. Sci. Rep..

[16] Galy B., Conrad M., Muckenthaler M. (2024). Mechanisms controlling cellular and systemic iron homeostasis. Nat. Rev. Mol. Cell Biol..

[17] Piperno A., Pelucchi S., Mariani R. (2020). Inherited iron overload disorders. Transl. Gastroenterol. Hepatol..

[18] Isidori A., Loscocco F., Visani G., Chiarucci M., Musto P., Kubasch A.S., Platzbecker U., Vinchi F. (2021). Iron Toxicity and Chelation Therapy in Hematopoietic Stem Cell Transplant. Transplant. Cell. Ther..

[19] Fernandez M., Lokan J., Leung C., Grigg A. (2022). A critical evaluation of the role of iron overload in fatty liver disease. J. Gastroenterol. Hepatol..

[20] Sawicki K.T., De Jesus A., Ardehali H. (2023). Iron Metabolism in Cardiovascular Disease: Physiology, Mechanisms, and Therapeutic Targets. Circ. Res..

[21] Martines A.M., Masereeuw R., Tjalsma H., Hoenderop J.G., Wetzels J.F., Swinkels D.W. (2013). Iron metabolism in the pathogenesis of iron-induced kidney injury. Nat. Rev. Nephrol..

[22] Ryan S.K., Ugalde C.L., Rolland A.S., Skidmore J., Devos D., Hammond T.R. (2023). Therapeutic inhibition of ferroptosis in neurodegenerative disease. Trends Pharmacol. Sci..

[23] Jiang X., Stockwell B.R., Conrad M. (2021). Ferroptosis: Mechanisms, biology and role in disease. Nat. Rev. Mol. Cell Biol..

[24] Dixon S.J., Lemberg K.M., Lamprecht M.R., Skouta R., Zaitsev E.M., Gleason C.E., Patel D.N., Bauer A.J., Cantley A.M., Yang W.S. (2012). Ferroptosis: An iron-dependent form of nonapoptotic cell death. Cell.

[25] Wu W., Chang S., Wu Q., Xu Z., Wang P., Li Y., Yu P., Gao G., Shi Z., Duan X. (2016). Mitochondrial ferritin protects the murine myocardium from acute exhaustive exercise injury. Cell Death Dis..

[26] Wu Q., Wu W.S., Su L., Zheng X., Wu W.Y., Santambrogio P., Gou Y.J., Hao Q., Wang P.N., Li Y.R. (2019). Mitochondrial Ferritin Is a Hypoxia-Inducible Factor 1alpha-Inducible Gene That Protects from Hypoxia-Induced Cell Death in Brain. Antioxid. Redox Signal.

[27] Gao M., Yi J., Zhu J., Minikes A.M., Monian P., Thompson C.B., Jiang X. (2019). Role of Mitochondria in Ferroptosis. Mol. Cell.

[28] Oh S.J., Ikeda M., Ide T., Hur K.Y., Lee M.S. (2022). Mitochondrial event as an ultimate step in ferroptosis. Cell Death Discov..

[29] Krainz T., Gaschler M.M., Lim C., Sacher J.R., Stockwell B.R., Wipf P. (2016). A Mitochondrial-Targeted Nitroxide Is a Potent Inhibitor of Ferroptosis. ACS Cent. Sci..

[30] Fang X., Wang H., Han D., Xie E., Yang X., Wei J., Gu S., Gao F., Zhu N., Yin X. (2019). Ferroptosis as a target for protection against cardiomyopathy. Proc. Natl. Acad. Sci. USA.

[31] Lyamzaev K.G., Panteleeva A.A., Simonyan R.A., Avetisyan A.V., Chernyak B.V. (2023). Mitochondrial Lipid Peroxidation Is Responsible for Ferroptosis. Cells.

[32] Maus M., Lopez-Polo V., Mateo L., Lafarga M., Aguilera M., De Lama E., Meyer K., Sola A., Lopez-Martinez C., Lopez-Alonso I. (2023). Iron accumulation drives fibrosis, senescence and the senescence-associated secretory phenotype. Nat. Metab..

[33] Burhans W.C., Heintz N.H. (2009). The cell cycle is a redox cycle: Linking phase-specific targets to cell fate. Free Radic. Biol. Med..

[34] Chapman J., Fielder E., Passos J.F. (2019). Mitochondrial dysfunction and cell senescence: Deciphering a complex relationship. FEBS Lett..

[35] von Zglinicki T., Nilsson E., Docke W.D., Brunk U.T. (1995). Lipofuscin accumulation and ageing of fibroblasts. Gerontology.

[36] Hohn A., Jung T., Grimm S., Grune T. (2010). Lipofuscin-bound iron is a major intracellular source of oxidants: Role in senescent cells. Free Radic. Biol. Med..

[37] Chio K.S., Reiss U., Fletcher B., Tappel A.L. (1969). Peroxidation of subcellular organelles: Formation of lipofuscinlike fluorescent pigments. Science.

[38] Terman A., Kurz T., Navratil M., Arriaga E.A., Brunk U.T. (2010). Mitochondrial turnover and aging of long-lived postmitotic cells: The mitochondrial-lysosomal axis theory of aging. Antioxid. Redox Signal.

[39] Lyamzaev K.G., Sumbatyan N.V., Nesterenko A.M., Kholina E.G., Voskoboynikova N., Steinhoff H.J., Mulkidjanian A.Y., Chernyak B.V. (2019). MitoCLox: A Novel Mitochondria-Targeted Fluorescent Probe for Tracing Lipid Peroxidation. Oxid. Med. Cell Longev..

[40] Lyamzaev K.G., Panteleeva A.A., Karpukhina A.A., Galkin I.I., Popova E.N., Pletjushkina O.Y., Rieger B., Busch K.B., Mulkidjanian A.Y., Chernyak B.V. (2020). Novel Fluorescent Mitochondria-Targeted Probe MitoCLox Reports Lipid Peroxidation in Response to Oxidative Stress In Vivo. Oxid. Med. Cell. Longev..

[41] Chernyavskij D.A., Pletjushkina O.Y., Kashtanova A.V., Galkin I.I., Karpukhina A., Chernyak B.V., Vassetzky Y.S., Popova E.N. (2023). Mitochondrial Oxidative Stress and Mitophagy Activation Contribute to TNF-Dependent Impairment of Myogenesis. Antioxidants.

[42] Malavolta M., Giacconi R., Piacenza F., Strizzi S., Cardelli M., Bigossi G., Marcozzi S., Tiano L., Marcheggiani F., Matacchione G. (2022). Simple Detection of Unstained Live Senescent Cells with Imaging Flow Cytometry. Cells.

[43] Fang S., Yu X., Ding H., Han J., Feng J. (2018). Effects of intracellular iron overload on cell death and identification of potent cell death inhibitors. Biochem. Biophys. Res. Commun..

[44] Atamna H., Nguyen A., Schultz C., Boyle K., Newberry J., Kato H., Ames B.N. (2008). Methylene blue delays cellular senescence and enhances key mitochondrial biochemical pathways. FASEB J..

[45] Gureev A.P., Sadovnikova I.S., Popov V.N. (2022). Molecular Mechanisms of the Neuroprotective Effect of Methylene Blue. Biochemistry.

[46] Porta E., Llesuy S., Monserrat A.J., Benavides S., Travacio M. (1995). Changes in cathepsin B and lipofuscin during development and aging in rat brain and heart. Gerontology.

[47] Nakano M., Oenzil F., Mizuno T., Gotoh S. (1995). Age-related changes in the lipofuscin accumulation of brain and heart. Gerontology.

[48] Zhang Q., Wang Q., Ding H., Hu C., Feng J. (2024). Ferroptosis Altered microRNAs Expression in HT-1080 Fibrosarcoma Cells Based on Small RNA Sequencing and Bioinformatics Analysis. Nutrients.

[49] Li Y., Wang B., Yang J., Liu R., Xie J., Wang J. (2023). Iron Overload Causes Ferroptosis but Not Apoptosis in MO3.13 Oligodendrocytes. Neurochem. Res..

[50] Wu W., Geng Z., Bai H., Liu T., Zhang B. (2021). Ammonium Ferric Citrate induced Ferroptosis in Non-Small-Cell Lung Carcinoma through the inhibition of GPX4-GSS/GSR-GGT axis activity. Int. J. Med. Sci..

[51] Kuang H., Sun X., Liu Y., Tang M., Wei Y., Shi Y., Li R., Xiao G., Kang J., Wang F. (2023). Palmitic acid-induced ferroptosis via CD36 activates ER stress to break calcium-iron balance in colon cancer cells. FEBS J..

[52] Deng L., Mo M.Q., Zhong J., Li Z., Li G., Liang Y. (2023). Iron overload induces islet beta cell ferroptosis by activating ASK1/P-P38/CHOP signaling pathway. PeerJ.

[53] Simcox J.A., McClain D.A. (2013). Iron and diabetes risk. Cell Metab..

[54] Jiang Z., Wang H., Qi G., Jiang C., Chen K., Yan Z. (2022). Iron overload-induced ferroptosis of osteoblasts inhibits osteogenesis and promotes osteoporosis: An in vitro and in vivo study. IUBMB Life.

[55] Wang Y.Q., Chang S.Y., Wu Q., Gou Y.J., Jia L., Cui Y.M., Yu P., Shi Z.H., Wu W.S., Gao G. (2016). The Protective Role of Mitochondrial Ferritin on Erastin-Induced Ferroptosis. Front. Aging Neurosci..

[56] Brunk U.T., Terman A. (2002). Lipofuscin: Mechanisms of age-related accumulation and influence on cell function. Free Radic. Biol. Med..

[57] Hohn A., Sittig A., Jung T., Grimm S., Grune T. (2012). Lipofuscin is formed independently of macroautophagy and lysosomal activity in stress-induced prematurely senescent human fibroblasts. Free Radic. Biol. Med..

[58] Ivy G.O., Kanai S., Ohta M., Smith G., Sato Y., Kobayashi M., Kitani K. (1989). Lipofuscin-like substances accumulate rapidly in brain, retina and internal organs with cysteine protease inhibition. Adv. Exp. Med. Biol..

[59] Konig J., Ott C., Hugo M., Jung T., Bulteau A.L., Grune T., Hohn A. (2017). Mitochondrial contribution to lipofuscin formation. Redox Biol..

[60] Biswal S., Barhwal K.K., Das D., Dhingra R., Dhingra N., Nag T.C., Hota S.K. (2018). Salidroside mediated stabilization of Bcl -x(L) prevents mitophagy in CA3 hippocampal neurons during hypoxia. Neurobiol. Dis..

[61] Frolova M.S., Surin A.M., Braslavski A.V., Vekshin N.L. (2015). Degradation of Mitochondria to Lipofuscin upon Heating and Illumination. Biofizika.

[62] Kohutiar M., Ivica J., Vytasek R., Skoumalova A., Illner J., Santorova P., Wilhelm J. (2016). Comparison of the effects of tert-butyl hydroperoxide and peroxynitrite on the oxidative damage to isolated beef heart mitochondria. Physiol. Res..

