# Supplementary material for: Exogenous Iron Induces Mitochondrial Lipid Peroxidation, Lipofuscin Accumulation, and Ferroptosis in H9c2 Cardiomyocytes
Source: Biomolecules. 2024 Jun 19;14(6):730. doi: 10.3390/biom14060730 (PMC11201805; doi:10.3390/biom14060730)
Supplement: Supplementary file 1 [file biomolecules-14-00730-s001.zip › biomolecules-3006169-supplementary.pdf]

Supplement

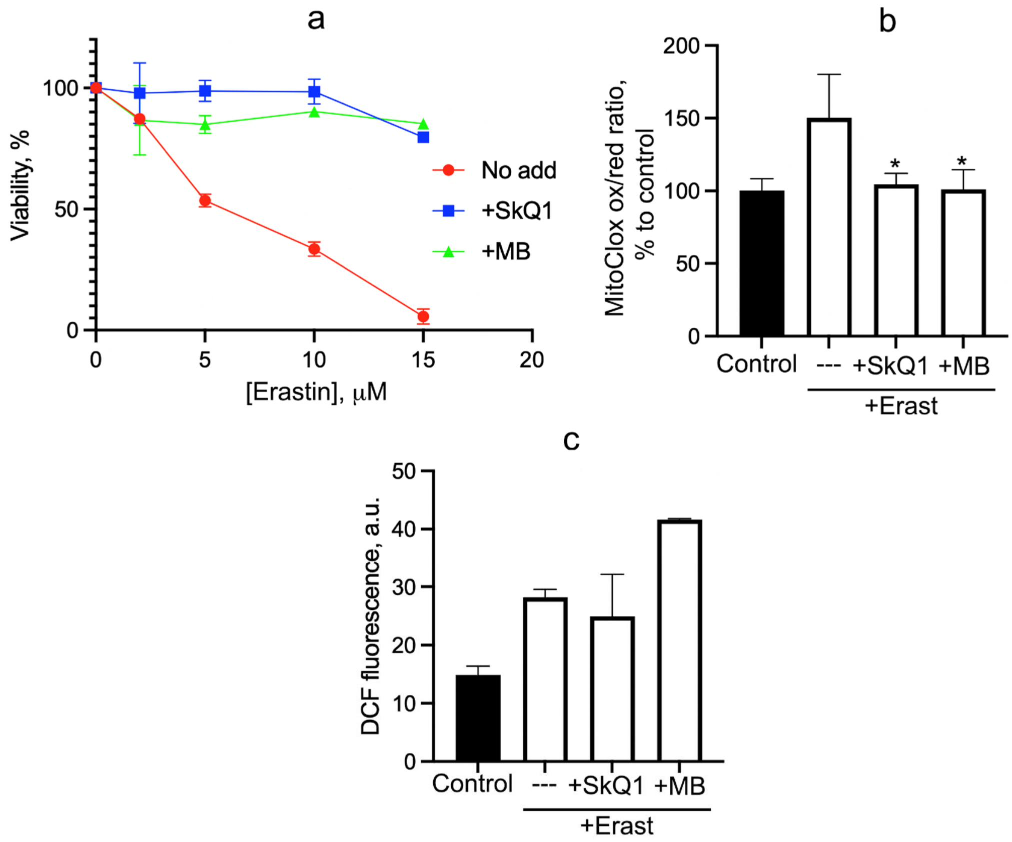

Suppl. Figure S1. Erastin-induced ferroptosis in H9C2. (a) Cells were incubated with different concentration of Erastin for 24h. Cell viability was measured using the CellTiterBlue reagent. 50 nM SkQ1 (blue line) or 250 nM Methylene Blue (green line) was added simultaneously with Erastin. (b,c) Cells were incubated as in (a) for 17h and stained with 100 nM MitoCLox for 1 h (b) or with 1.8 μM CM-H2DCFDA for 30 min (c). Ratio of green/red fluorescence was measured and analyzed (b). Mean values of fluorescence are presented. $p < 0.05$(*) - the significance of the difference between samples treated with Erastin and other samples.
